# Corruption and informal sector households’ participation in health insurance in Sierra Leone

**DOI:** 10.1371/journal.pone.0281724

**Published:** 2023-04-13

**Authors:** Mireia Jofre-Bonet, Joseph Kamara, Alice Mesnard

**Affiliations:** 1 Department of Economics, School of Policy & Global Affairs, City University of London, London, United Kingdom; 2 Department of Research, Office of Health Economics, London, United Kingdom; 3 Department of Social Health Insurance, National Social Security and Insurance Trust (NASSIT), Freetown, Sierra Leone; University of Bologna, ITALY

## Abstract

Lack of credibility and trust in fund managers has been highlighted as one of the key reasons why people do not join health insurance schemes in low- and middle-income countries, especially in Africa. This work investigates the impact of corruption on households’ willingness to participate and pay for health insurance in Sierra Leone. A discrete choice experiment (DCE) method was used to elicit households’ willingness to participate in a health insurance scheme with different attributes. The data were collected from 1458 representative households working in the informal sector of the Northern and Western regions. We explore the relationship between household characteristics and experienced (respectively, perceived) corruption with binary and ordered logit models. We use a Mixed Logit model to estimate the association between corruption and participation in a Health Insurance Scheme (HIS) and households’ willingness to pay for a HIS. We find that corruption decreases participation in a public HIS and the willingness to pay for it. Our results highlight the perverse spillover effects of corruption. Not only does corruption hinder the effectiveness of healthcare systems and, thus, worsen health outcomes. It also undermines the willingness to pay for them, jeopardizing the sustainability of healthcare systems in the countries that need them most.

## Introduction

A fundamental problem developing countries face is corruption creating barriers to development [1,2]. Corruption has made healthcare particularly costly or unaffordable for most of the population, damaged patient care and demotivated healthcare workers [3]. This has led to inefficient health systems with poor quality services, inequitable access, and inadequate funding [1,4,5]. Sadly, in these countries, corruption often is perceived as a spillover effect of government intervention that can negatively affect the provision of health care services [6].

Considerable evidence supports the point that unofficial payments are deeply entrenched in markets for healthcare in developing countries [5,7]. In addition, studies show that health sector corruption contributes significantly to developing countries’ poor health situation [7–12]. Even though corruption is salient, estimating its overall cost in the health sector is challenging for several reasons: it is difficult to distinguish between corruption, inefficiency, and honest mistakes; record-keeping is often neglected; and the health sector has many stakeholders, enabling corruption to proliferate to various sub-sectors.

Health officials engage in corruption for various reasons, but key amongst them are (i) opportunities generated by monopoly in service delivery, discretion in decision making, poor accountability and transparency; (ii) a conducive environment where public service values are eroded, and corruption is perceived as a condition to success; (iii) low salaries, personal financial debts and similar pressures [13]. Lewis describes that, in developing countries, medical staff are involved in under-the-table corruption because of the low and irregular payment of their salaries, lack of government action in the health care system, and the culture of gifts [4]. Lastly, the presence of information asymmetry in the health system, its complexity, and the inherent uncertainty in healthcare markets favour the emergence of corruption [13,14].

Akin to many other developing countries, corruption is one of the most important factors to have hindered growth in the health sector of Sierra Leone. The corruption in Sierra Leone’s health sector ranges from demanding bribes for basic services to large-scale misuse of public goods for private gain by public officials [15]. In the Transparency International Corruption Perceptions Index 2017, Sierra Leone scored 30 out of 100 (where 0 is considered highly corrupt and 100 the opposite), ranking 130 out of 175 countries. A local survey by the Anti-Corruption Commission (ACC) in Sierra Leone in 2010 shows that most Sierra Leoneans have experienced corruption in one way or another, with 94% classifying it as a problem [16]. In March 2013, the ACC indicted 29 National Health Sector Support Project (NHSSP) officials at the Ministry of Health and Sanitation for various corruption offences regarding misuse of the Global Alliance for Vaccines and Immunization (GAVI) funds. The various charges amounted to $2,436,921 [16,17]. Pieterse and Lodge opine that corruption scandals have plagued the Free Health Care initiative in Sierra Leone [3]. They quote an Amnesty International report of 2009 showing that, in most areas, staff would unilaterally and illegally charge fees and keep the money.

Moreover, the 2018 Afro barometer survey in Sierra Leone reveals that many Sierra Leoneans report delays and difficulties accessing care at public hospitals and clinics. About half say they pay a bribe to access it [17]. The huge systemic corruption in Sierra Leone explains why up to a third of the money given to fight Ebola remains unaccounted for [15]. With an adult literacy rate of 40%, many people are not empowered to stand up for their right to free healthcare and are often not even aware that the charges they are being asked to pay are unauthorized [18]. Furthermore, there is inevitably a fear of reporting corruption as people fear being harassed or excluded on their next visit.

Several studies agree that corruption within the health sector is severe and must be addressed urgently; otherwise, the poor will continue to get poorer and have a shorter life expectancy [13,19–21]. The effect of corruption is likely to have a greater burden on those who are impoverished and cannot afford to pay bribes or seek private alternatives. The Public Affairs Centre survey reveals that as much as 38% of total hospital expenses borne by households are in the form of bribes, and some 17% of households claim to have made unofficial payments to public hospitals [22]. Moreover, people’s perception of corruption in the health sector strongly correlates with input overpricing and unofficial payments [23].

In Sierra Leone, like in many low- and middle-income countries, most of the adult population works in the informal sector, where activities are typically unrecorded and exist in the narrow space between legality and illegality [24]. The informal sector emerges as a means to avoid taxes, environmental norms and labour laws [24,25]. With only about 20% of adults having bank accounts in Sierra Leone, formal transaction costs are replaced by informal payments and bribes. Informal employment is unregistered, and workers are habitually paid lower wages and receive fewer benefits, such as social security, than those who work in formal employment [25].

However, there is not much literature on the impact of corruption on the uptake of (usually voluntary) state-subsidized or social health insurance schemes for the informal sector in low- and middle-income countries. To be meaningful, the estimation of the uptake drivers must overcome the problem of self-selection into insurance, which is not always possible and/or achieved. A review of the literature on this subject reveals that the uptake of insurance schemes is lower than expected [26]. There is evidence that the low participation in Health Insurance Schemes (HIS) might be partly demand-driven and, therefore, could be addressed through an appropriate HIS design [27]. However, several studies point out that low demand for HISs might be caused by the lack of credibility of public services due to corruption [28]. The healthcare sector’s degree of corruption or, at least, the informal sector’s perception or experience of corruption is rarely captured in surveys on participation in HIS. Thus, the impact of corruption in the healthcare sector on participation and willingness to pay in HIS remains unanswered.

This paper aims to shed light on this question. Our purpose is twofold: first, we study the relationship between corruption, both perceived and experienced in the health sector, and the characteristics of households working in the informal sector. Second, we assess the impact of corruption in the health sector on their participation and willingness to pay for a HIS. This paper is structured as follows. Section 2 outlines the methodology and Section 3 the results. Section 4 discusses them and Section 5 concludes.

## Methodology

### Study area

Our study takes place in the Northern and Western regions of Sierra Leone, which cover both a poorer region (the North) and a richer one (the West).

Health care in Sierra Leone is provided by a mixture of government, faith-based (private not-for-profit) and private (for-profit) health providers. The entire healthcare system is divided into three tiers of care: Peripheral Care Units (PHUs), which also have community health posts (CHPs); secondary care, which includes district hospitals; and tertiary care, which has both regional hospitals and referral hospitals. Most secondary and tertiary care hospitals are in the western region.

Sierra Leone has one of the weakest health systems and faces serious challenges. These include heavy disease burden, chronic underfunding coupled with a lack of human resources for health (both in quantity and quality). The health indicators of Sierra Leone are poor, with very high rates of female mortality and morbidity when giving birth and very high infant and under-five mortality rates [29]. Communicable diseases are the leading cause of death in Sierra Leone, with Malaria accounting for 38% [30]. Out-of-Pocket payments are very high, contributing to the high cost of health care and the inability of the population to financially access services.

The Free Healthcare Initiative was introduced in 2010 to abolish user fees for all pregnant and lactating women and children under-five to address this problem. The government launched the Sierra Leone Social Health Insurance (SLeSHI) Scheme in 2018 to improve financial access to health care. This scheme was not yet established when conducting our survey, but there was awareness surrounding the project.

This study aimed to explore the potential impact of corruption on the scheme’s feasibility. The SLeSHI was conceived as a contributory, mandatory scheme for every Sierra Leonean in the country. However, other sources of income were proposed, such as budgetary support, the introduction of a health insurance levy from the goods and services tax, etc. The contribution is twofold: formal sector workers pay 2% of their basic pay, and their employer pays 4%. The informal sector workers pay a flat fee of 15,000 Sierra Leonean Leones (approximately $3.15). For a start, the scheme provides benefits in Primary Health Care (PHC) services and selected secondary care services, including minor operations. This was decided on the basis that about three-fourths of the disease burden is around PHC services; hence starting with them will reduce the disease burden in the country.

### Discrete choice experiment

The data were obtained by running a Discrete Choice Experiment (DCE) conducted in the Northern and Western regions in 2016 [31,32]. A DCE is a survey-based data collection method used to elicit preferences over a set of characteristics of a product, service, or intervention [33]. In our case, a DCE was used to learn households’ preferences regarding a HIS by allowing them to choose to participate or not in a set of HISs described by their distinct attributes and their levels, including cost. A DCE procedure allows for establishing trade-offs between traits and levels of attributes [33].

After an in-depth literature search, we identified ten attributes relevant to a HIS. We sent these to 50 households to rank them in order of importance. The question asked in the pre-test survey reads: *"Assuming a national health insurance scheme is to be introduced in the country*, *what are the key factors you will take into consideration before joining the said scheme*, *and from the list of attributes shown*, *please tick according to your order of preference"*. The four most important attributes highlighted by the households were coverage, waiting time, choice of healthcare provider and cost.

As one of the key objectives of this study is to estimate households’ willingness to pay (WTP) for health insurance, the choice of cost among the key attributes is critical. We ensured that the chosen attributes were also plausible, quantifiable, and easily recognizable for the respondents. In line with the preceding argument and WTP studies in low- and middle-income countries [26], we assigned three levels to each attribute we chose. The final four attributes and their levels used are shown in Table 1 below.

**Table 1:** Attributes and levels used in the DCE.

Attributes	Attribute Levels	Description
Coverage	Simple Moderate Comprehensive	Primary Health Care Diseases and Minor Operations Simple coverage plus Secondary Health Care and Major Operations Moderate coverage plus Tertiary Health Care
Waiting Time	45 Minutes 60 Minutes 90 Minutes	The length of time one has to wait before seeing medical personnel
Choice of Provider	Private Public Faith-Based	Health centres and hospitals owned and operated privately Health centres and hospitals owned and operated publicly Health centres and hospitals owned and operated by faith-based organizations
Cost/Premium	4000SLL (0.54USD) 6000SLL (0.81USD) 10000SLL (1.35USD)	The monthly premium members will pay for the scheme

Notes: The exchange rate used right through this work is that in November 2016; $1 = SLL 7,400, where SLL stands for the Sierra Leone currency.

A generic (unlabeled), forced-choice, efficient, experimental design was employed in this study. We chose a forced-choice (no outside option) design to maximize the number of usable responses and obtain robust preference estimates. Under the assumption that the HIS could be compulsory, gathering information on choice ranking seemed appropriate.

We generated the choice HIS profiles using an experimental design [34,35]. The four attributes and three levels resulted in 81 hypothetical HIS profiles. To ensure the cognitive burden of respondents is reduced to a workable size, we used a fractional factorial design (FFD) to reduce the number of profiles to a manageable number without losing the chance of estimating the main effects. The efficiency of the experimental design compared to the full design was verified as 91.7%, using Street and Burgess online design [36]. The software SPSS was used to construct the experimental design. The FFD generated a total of 18 choice profiles, subsequently divided into two blocks of 9 choice sets each. Hence, each household was presented with ten-choice questions: nine for the main DCE and one to test the level of understanding of the questions.

An additional set of questions were included in the survey to collect information on the socio-demographic characteristics of households, their perception of corruption, their current health status and how they finance their health. Interviewers were recruited locally and trained.

### Sampling techniques and sample size

Statistics Sierra Leone (SSL) designed the sample and selected locations for this study based on recent pre-census data containing settlement names, population, and household sizes. A two-stage stratified random sampling method was used to identify the households. The first stage involved stratifying the population by region/district, and the second by rural and urban location in each district. The purpose was to have a representative sample of informal sector households in both villages (rural areas) and major towns (urban areas). Since the decision to purchase health care is more often a household decision rather than an individual one, the household was chosen as the unit of reference.

SSL calculated the sample size of 1670, considering an informal sector population of 1,380,110 individuals, with the objective to achieve a 95% confidence interval around participation in the HIS and a margin of error of 2.4% [37]. Due to failure to pass the dominance test (understanding of the questions) and incomplete data during the questionnaire administration, 1,458 households’ data were used for the final analysis, which provided 39,366 observations for the analysis of nine choice sets with three alternatives. Eight predominant informal sector activities were chosen for the survey: Petty Trading, Subsistence Farming, Commercial Bike Riding (Okada), Cattle-rearing, Fishing, Tailoring, Mining, and Quarrying. The econometric analysis was done using STATA version 13.1.

### Variables

The answers to our survey generated two measures of corruption: the first reports if a household has *perceived corruption* within the health sector on a scale of 1 (not corrupt) to 4 (highly corrupt), which we label as *perccorr*. The second indicates experienced *corruption*, i.e., whether households had paid for treatments at public hospitals or health centres supposed to be free under the Free Health Care (FHC) treatment Act (2010) for children under five years old, pregnant women and lactating mothers. Labelled *fhccorr*, the latter takes value 1 if households say they paid for treatments and services supposed to be free and 0 otherwise. The reasons given by households in our sample for paying for treatments that should have been free included being unaware that these services were free; the medical personnel requested the payment; they paid willingly, and other less important reasons. In the literature, paying for treatments that should be free is an indicator of actual corruption in the healthcare system [38].

Table 2 defines all household characteristics included in the analysis, which are likely to be associated with different experiences of corruption and different levels of perceived corruption. These traits are household per capita income (hereafter HHPCI); economic activity of the head; the age of the head; years of school of the head; distance to a healthcare centre; household size; diseases experienced (Malaria or typhoid fever in the previous three months); location type (rural or urban); receives remittances from abroad; and, whether the head listens to the radio and reads newspapers regularly.

**Table 2:** Variable definitions.

Variable	Definition
Participation	Reported household’s decision to participate in HIS. Equal to 1 when answered Yes to participation and 0 otherwise.
FHC Corruption (*fhcorr*)	Whether the household reports that they pay medical expenses for a member in any of these categories: under-fives, pregnant women, and lactating mothers. Equal to 1 if the answer is Yes and 0 otherwise.
Corruption Perception (*perccorr*)	Household’s perception of corruption in the health sector: (1) Not corrupt (2) Fairly corrupt (3) Corrupt (4) Very corrupt.
Cost	Amount to be paid for the HIS: 4,000, 6,000 & 10,000 SLL.
Coverage^1^	Coverage of the scheme: Simple, Moderate & Comprehensive.
Waiting Time	Waiting time to see a doctor/nurse: 45, 60 and 90 minutes.
Public Provider	Health services offered by a public provider = 1, 0 otherwise.
Faith-Based–Provider	Health services offered by a faith-based provider = 1, 0 otherwise.
Household Per Capita Income (HHPCI)	Calculated household per capita income in Sierra Leonean Leones (SLL) Derived variable obtained dividing household income by the square root of the household size.
Petty Trading	Reported type of informal sector = 1; 0 otherwise.
Subsistence Farming	Reported type of informal sector = 1; 0 otherwise.
Okada	Reported type of informal sector = 1; 0 otherwise.
Cattle-rearing	Reported type of informal sector = 1; 0 otherwise.
Fishing	Reported type of informal sector = 1; 0 otherwise.
Tailoring	Reported type of informal sector = 1; 0 otherwise.
Mining	Reported type of informal sector = 1; 0 otherwise.
Quarrying	Reported type of informal sector = 1; 0 otherwise.
School	Reported whether household went to school = 1; 0 otherwise.
Distance to Health Centre	Distance in miles from village to the nearest health centre.
Age of Household Head	Age of household head.
Household Size	Number of members in the household at the time of the interview.
Diseases	Whether any household member has suffered from malaria or typhoid fever in the three months prior to the interview; yes = 1; 0 otherwise
Urban Setting	Household location: rural or urban; Urban = 1, Rural = 0.
Remittance	Whether household receives remittance from abroad; Yes = 1, 0 otherwise
News	Whether the household head listens to the radio or reads newspapers; Yes = 1; 0 otherwise
Interactions:	
Fhcorr*public	Interaction of experienced corruption and HIS services offered by a public provider; Yes = 1; 0 otherwise.
Perccorr*public	Interaction of perceived corruption and HIS services offered by a public provider; Yes = 1; 0 otherwise

Notes: Moderate coverage is used as the definition of coverage in this work.

### Estimation strategy

We propose three different models to answer the questions we deem of interest. The first two models estimate how household characteristics are associated with the household having experienced actual corruption (*fhcorr*), *i*.*e*., households paying for treatments that should be free, and with the likelihood of the household perceiving corruption (*perccorr)*. Since *fhccorr* is a binary variable and *percorr* is an ordered categorical variable with four levels, we use a logit model and an ordered logit model, respectively, to estimate their association with household characteristics. The third model examines the heterogeneity in household preferences for different HIS attributes. It examines how the household’s experience of corruption in the health sector influences the type of HIS choice.

To do so, we use a DCE relying on the literature summarised by Ryan and Gerard (2003). Given HIS attributes and the levels of their characteristics, the estimation is based on drawing independent samples of potential HIS scenarios from the full factorial set. In line with the Random Utility Theory, households decide based on comparing the indirect utility they associate with different HIS choices [39]. We note *U*_*ij*_ = *v*_*ij*_+*ε*_*ij*_ the indirect utility to individual *i* of choosing HIS *j*, *v*_*ij*_ being its *measurable* component, and *ε*_*ij*_ the *unobservable random* component or error term and specify the utility associated with household *i’s* participation in the HIS *j* as a linear function of its attributes written as:

$$U_{ij}=\beta_{0}+\beta_{1i}{Cost}_{j}+\beta_{2i}{Cov}_{j}+\beta_{3i}{Wait}_{j}+\beta_{4i}{Pub}_{j}+\beta_{5i}{Cont}_{j}+\beta_{6i}{corr\_pub}_{ij}+\varepsilon_{ij},$$

where *β*_0_ is the intercept and *Cost*_*j*_, *Cov*_*j*_, *Wait*_*j*_, *Pub*_*j*_, and *Cont*_*j*_ are attributes of HIS j. *Cost* is the cost of the scheme; *Cov* takes value 1 if HIS j provides moderate coverage; *Wait* is the waiting time associated with HIS j; *Pub* takes value 1 if HIS j is offered by a public provider, and *Cont* takes value 1 if HIS j belongs to a faith-based provider.

We model household i choice of HIS A over B if and only if *U*_*iA*_ > *U*_*iB*_, which means (*v*_*iA*_−*v*_*iB*_)>(*ε*_*iB*_−*ε*_*iA*_). Assuming a specific distribution of the error term, this yields a probabilistic model: $P(i\;choosing\;HIS\;A\;over\;B)=P({({\varepsilon_{iB}-\varepsilon }_{iA})<(v}_{iA}-v_{iB})$).

To allow for unobserved heterogeneity of preferences, we estimate the DCE using Mixed Logit Models (MXL). It also allows for individuals’ repeated observations and relaxes the assumption of the independence of irrelevant alternatives, i.e., that the introduction or removal of one choice in a conditional logit model has no effect on the proportion of probability assigned to each of the other choices [39–43].

Previous work on WTP for HIS suggests that households favour public providers [32]. Here, we test whether this is the case independently of corruption and when a household has experienced/perceived a high level of corruption. To do so, equation (1) includes two terms, a term *pub*_*ij*_, to capture the preference for public providers and a term, corr*pub_*ij*_, which is the interaction of the household i’s (experienced or perceived) corruption and the attribute of a HIS offered by a public provider. The latter captures whether corruption experience (or perceptions) affect household participation in HIS offered by a public provider. Note that the introduction of the interaction enables the slope coefficients to differ between subgroups while preserving the attribute level distribution variability [41].

In a DCE model, coefficients indicate the relative importance of each attribute. The sign of a coefficient reflects whether the attribute positively or negatively affects utility. The trade-offs that respondents are willing to make between attributes can be estimated by the ratios of the coefficients [33]. For instance, the ratio of the coefficients of coverage versus public provider, Cov/Pub, represents an estimate of how much additional coverage the respondent is willing to accept or forsake in order to have a public provider as opposed to another type. The coefficient associated with the variable Cost can be used to estimate the WTP for another attribute. For example, the WTP to avoid having to wait can be obtained by dividing the coefficients associated with waiting by the negative of that associated with cost, i.e. -*β*_3i_/*β*_1i_. To assess whether a particular household characteristic subtracts or adds to the WTP for an attribute, one can also introduce interaction terms in the regression model. Accordingly, *β*_6i_/*β*_1i_ measures by how much the household’s experience of corruption changes its WTP for a HIS offered by a public provider.

As summarised above, we estimate the association of perceived corruption with household characteristics using an ordered logit model, an extension of the binary logistic model for cases where the dependent variable takes ordered categorical values. Using the Brant test, we assess the odds proportionality assumption of the estimated ordered logit model. The Brant test compares the separate fits to the binary logistic models with common regression parameters underlying the overall model [44].

## Results

This section presents some descriptive statistics, the estimates of the correlations of household characteristics with actual (or perceived) corruption and the effects of HIS’ attributes on the likelihood of participation in given schemes.

### Data summary and descriptive statistics

Table 3 below presents descriptive statistics of the socio-demographic characteristics of the sample. To motivate the analysis, we present the descriptive statistics of the means and standard deviations of the main variables of interest by subgroups: first, for those that had experienced corruption and those who had not; second, for those that declared perceiving corruption, including a high level of corruption. We provide t-test statistics comparing the means between those that did and did not experience (perceive) corruption.

**Table 3:** Descriptive statistics.

Variable	Experienced corruption			Perceived high Corruption		
	Yes	No	Significant difference*	Yes	No	Significant difference*
	Mean (SD)	Mean (SD)		Mean (SD)	Mean (SD)	
**Petty Trading**	24.90%	28.50%	8.0684	25.50%	27.50%	4.1685
	0.432	0.452	0.00000	0.436	0.447	0.00000
**Sub. Farming**	24.50%	16.10%	20.4055	20.60%	19.40%	-2.6952
	0.43	0.368	0.00000	0.405	0.396	0.007
**Okada**	20.50%	23.90%	8.0544	23.30%	22.10%	-2.6076
	0.404	0.426	0.00000	0.423	0.415	0.0091
**Cattle-rearing**	5.80%	4.30%	-6.8354	0.49%	5.00%	0.4846
	0.234	0.202	0.00000	0.215	0.217	0.628
**Fishing**	6.10%	9.10%	11.3962	9.50%	7.20%	-7.2378
	0.24	0.288	0.00000	0.293	0.258	0.00000
**Tailoring**	11.30%	9.90%	-4.4876	10.20%	10.60%	1.2269
	0.316	0.298	0.00000	0.303	0.308	0.2199
**Mining**	3.60%	3.80%	0.9171	2.90%	4.00%	5.5786
	0.186	0.191	0.3591	0.168	0.196	0.00000
**Quarry**	3.30%	4.40%	5.6783	3.20%	4.20%	5.145
	0.178	0.205	0.00000	0.175	0.201	0.00000
**Distance to Health Centre**	2.13	2.16	1.3371	2.1	2.16	2.415
	2.468	2.686	0.1812	2.235	2.721	0.0157
**Urban setting**	44.80%	49.60%	9.4905	44.20%	48.90%	8.3994
	0.497	0.5	0.00000	0.45	0.5	0.00000
**Age of Household Head**	44.62 14.56	42.7 12.07	-11.3747 0.0000	53.6 13.07	45.3 15.14	-14.5718 0.0000
**School**	62.6%	69.5%	19.2004	69.8%	63.5%	-11.7388
	1.423	1.381	0.0000	1.36	1.421	0.00000
**Remittances**	21.50%	12.80%	-22.5078	19.40%	15.50%	-9.0874
	0.411	0.334	0.00000	0.396	0.362	0.00000
**Diseases**	74.60%	72.60%	-4.5391	75.70%	72.60%	-6.5423
	0.435	0.446	0.00000	0.429	0.446	0.00000
**News**	85.60%	77.60%	-20.7589	86.90%	78.80%	-20.1837
	0,351	0.417	0.00000	0.337	0.409	0.00000
**HHPCI**	153,900	159,600	10.57	154,942	157,954	5.07
	51,700	56,200	0.00000	52,249	55,166	0.00000
**Number of households**	957	501		923	535	

Notes: This table contains the means, standard deviations, and test for equality of proportions (categorical variables) or means (continuous) for those that experienced corruption versus those who did not; and those that perceived corruption or high corruption versus not. *The tests are based on Chi-Square distribution for categorical variables and T-Student distribution for continuous ones (distance, age, and income).

As shown in Table 3, most of the sample were engaged in the economic activity of Petty Trading (24.9% and 25.5% of those experiencing or perceiving high corruption, respectively), Subsistence Farming (24.5%, 20.6%), or Okada Riding (20.5%, 23.3%). The weight of Tailoring is significant (11.3%, 10.2%), while Fishing (6.1%, 9.5%), Mining (3.6%, 2.9%), and Quarrying (3.3%, 3.2%) are less prevalent. Between 44% and 49% of the sample live in an urban setting, and 15% to 21% receive remittances from abroad. The average age of the head of household oscillates between 43 to 54 years, and about 63% to 70% have attended school. The percentage of the sample having experienced bad health is about 70%. We test for the differences in means of those that answer they have experienced (perceived high level of) corruption and those that respond negatively. Curiously, all means are significantly different except for those working in Mining (for experienced corruption), and Cattle-rearing and Tailoring (for the perception of high levels of corruption). The distance to a health care centre is not different by subgroups (about 2km) for both proxies of corruption.

Households in Subsistence Farming, Cattle-rearing, and Tailoring, and lower in Petty Trading, Okada, Fishing, Quarry have statistically significant higher rates of having experienced corruption. Households having experienced corruption receive remittances more often, experienced bad health in the last three months and read the news. Moreover, they tend to have older heads who have attended school less often and live less frequently in urban settings. The differences for those perceiving high levels of corruption follow the same pattern, except that a higher proportion of those in Mining has not perceived corruption. At the same time, there is no difference for those in Cattle-rearing and Tailoring. The heads that have attended school are more likely to perceive high levels of corruption.

Other statistics of interest (not shown in tables) are the following: when the households sampled were asked why they paid for free services (FHC), 52% responded that they were asked to pay, 38% were unaware they should not pay, while another 6% said that they paid willingly. In terms of the type of treatment households sought when faced with a health shock, the respondents favoured predominantly health centres (48%) and hospitals (24.7%), followed by pharmacies (13.1%) and drug peddlers (8.1%), self-treatments (2.7%) and traditional treatments (2.5%).

### Relationship between corruption and household characteristics

This section studies the relationship between actual corruption (fhcorr = 1) and household characteristics using a logit model. Table 4 below reports the estimates of the coefficients, the corresponding odds ratio point estimate as well as its 95% confidence interval.

**Table 4:** Relationship between experienced corruption and household characteristics–logit.

Dependent variable:	FHC^1^ Corruption (*fhcorr)*			
Variables	Coefficients (SE)	Odds Ratio (SE)	Confidence Interval of Odds Ratio	
**Petty Trading** ^**2**^ **Subsistence Farming** **Okada** **Cattle-rearing** **Fishing** **Tailoring** **Mining** **HHPCI** **Distance to Health Centre** **Schooling** ^**3**^ **Urban Setting** ^**4**^ **Age of Household Head** **Diseases** ^**5**^ **Remittances Received** ^6^ **News** ^**7**^ **Constant**	0.28921 (0.07075)*** 0.59757 (0.07335)*** 0.36694 (0.07199)*** 0.66548 (0.08674)*** -0.04902 (0.08124) 0.48688 (0.07728)*** 0.34541 (0.09349)*** -0.41695 (0.03125)*** -0.02889 (0.00495)*** -0.35640 (0.03087)*** 0.00479 (0.02824) -0.00479 (0.00122)*** 0.18261 (0.03079)*** 1.62e-06 (1.09e-07)*** 0.52638 (0.03422)*** 3.88735 (0.37462)***	1.275595 (0.0900)*** 1.899158 (0.1417)*** 1.403977 (0.1005)*** 1.937981 (0.1685)*** 0.8799988 (0.0710) 1.552488 (0.1194)*** 1.448887 (0.1358)*** 0.9999981 (2.64E-07)*** 0.9843164 (0.0051)*** 0.8862225 (0.0088)*** 1.086989 (0.0336)** 1.000595 (0.0012) 1.215362 (0.0364)*** 1.667608 (0.0582)*** 1.632003 (0.0557)*** 0.5351681 (0.0561)***	1.110786 1.64077 1.220166 1.634304 0.751318 1.335237 1.205811 0.9999976 0.9743357 0.8691952 1.023019 0.9983278 1.14605 1.557333 1.526346 0.4357277	1.464858 2.198237 1.615477 2.298086 1.030719 1.805087 1.740963 0.999998 0.994399 0.903583 1.154958 1.002867 1.288867 1.78569 1.744975 0.657302
No of households No of observations LR Chi2 (15) Prob >Chi2 (k-1) Pseudo R2 Log-likelihood	1458 26226 1144.29 0.0000 0.0318 -17412.088			

Notes: 1.FHC stands for Free Health Care Corruption

2. The reference sector is Quarrying

3. No School is the base

4. Rural is the reference location used

5. No Disease, such as Malaria or typhoid in the previous three months, is the reference

6. Not receiving remittances is reference

7. Not listening to the news is the reference

Significance levels indicated by stars

*** p<0.01

** p<0.05

* p<0.1.

All variables in Table 4, apart from the economic activity of Fishing and the age of the head of household, are statistically significant. Thus, the probability of a household paying for health services that should have been free is significantly and positively correlated to whether the household is receiving remittances (odds ratio of 1.66), suffering from Malaria or typhoid disease (1.2), living in an urban setting (1.08), and the household head listening to the news (1.63). On the other hand, there is a negative correlation with distance to the health centre (0.97) and income per capita (0.99).

With respect to the occupation of the head of the household, the odds of answering yes to fhcorr is above one for households whose primary economic activity is either Petty Trading (1.27), Subsistence Farming (1.89), Okada (1.40), Cattle-rearing (1.93), Tailoring (1.55), and Mining (1.44) compared to households in the Quarrying sector (the reference), i.e., households involved in those sectors are more likely to have paid for services that should be free than those in Quarrying.

### Perceived corruption and household characteristics

Households were also asked to choose how they perceive corruption in the health sector on a scale from 1 to 4 (not corrupt to highly corrupt). This section reports the results of the ordered logit model we use to estimate the relationship between perceived corruption and household characteristics. Table 5 below presents the odds ratio estimates and their 95% confidence interval.

**Table 5:** Relationship between perceived corruption and household characteristics—ordered logit.

Dependent variable:	Corruption perception (*perccorr*)		
Variables	Odds Ratio (SE)	95% Confidence Interval	
Petty Trading Subsistence Farming Okada Cattle-rearing Fishing Tailoring Mining	2.190815 (.1109565) *** 3.328549 (.1801753) *** 2.380204 (.1217928) *** 2.356654 (.1498508) *** 4.1264 (.2394831) *** 3.517035 (.1974601) *** 2.169015 (.1471622) ***	1.98379 2.993499 2.153074 2.080517 3.682734 3.150554 1.898938	2.419446 3.701099 2.631294 2.669443 4.623516 3.926147 2.477505
HHPCI Distance to Health Centre Schooling* Urban* Age of HH Head Diseases* Remittances* News*	0.9999942 (2.289e-07) *** 0.8919685 (.0033515) *** 1.18815 (.0087903) *** .7909168 (.0180362) *** 1.005868 (.0008911) *** 1.397147 (.0303915) *** 1.043355 (.0262499) * 1.39382 (.0346867) ***	.9999936 .8854238 1.171046 .7563448 1.004123 1.338832 .9931542 1.327466	.9999948 .8985616 1.205504 .8270691 1.007616 1.458001 1.096094 1.46349
/cut1 /cut2 /cut3	-0.890113 (0.0753633) .8360194 (.0749496) 2.080758 (.075536)	-1.037822 .6891209 1.93271	-.7424037 .9829178 2.228806
Number of Households Number of Observations LR Chi2(15) Prob > chi2 Pseudo R2 Log-Likelihood	1458 39366 3516.22 0.0000 0.0341 -49792.761			

Notes: * See the references of categorical variables in the notes of Table 4.

Significant levels indicated by stars

*** p<0.01

** p<0.05

* p<0.1.

From Table 5, we conclude that working in all occupations substantially increases the likelihood of perceiving corruption compared to being in Quarrying, especially in Subsistence Farming (odds ratio of 3.32), Fishing and Tailoring (4.12 and 3.51, respectively). Having attended school (1.18), being an older head of household (1.0), an experience of bad health (1.39), and reading the news (1.4) make it more likely to perceive a high level of corruption. The odds ratio of receiving remittances is slightly above one (1.04) but only significant at 10% level. The odds ratio of the income per capita is statistically significant but very close to 1 (0.99). The distance to the nearest health centre (0.89) and living in an urban setting (0.79) are negatively correlated to perceiving high corruption levels.

Although we checked for the robustness of our results using an ordered probit model (results available upon request), they need to be interpreted with caution for two reasons. Firstly, it is difficult to compare the levels of perception across individuals based on a 1 to 4 scale as each individual may have a different reference system. This concern would be attenuated if for example we could use a fixed effects regression using longitudinal data, which we do not have. Secondly, a Brant test rejects the parallel regression assumption (as it is common, however, for this test, to reject the Null in real world situations). Nonetheless, the results suggest interesting patterns of correlations between household characteristics and their experience of corruption. Most variables have correlations of the same sign whether we use experienced corruption levels (as in Table 4) or perceived corruption levels as dependent variables (Table 5).

### Corruption and households’ participation in health insurance

We now turn to study the relationship between corruption and households’ preferences over HIS, for which we estimate a Mixed Logit model (MXL). To do so, we use 500 Halton draws for each sampled household to generate their simulated probability [27].

Table 6 below presents the estimation results of the MXL model for the full sample. Model (1) uses the variable fhcorr as a proxy for experienced corruption, whereas Model (2) uses the variable perccorr, measuring the household’s perceived level of corruption. The mean coefficients of the estimates are presented alongside their standard deviations.

**Table 6:** The Impact of corruption on households’ participation in health insurance-mixed logit.

	Participation in Health Insurance Model (1) using experienced corruption		Participation in Health Insurance Model (2) using perceived corruption	
Variables	Mean (SE)	SD (SE)	Mean (SE)	SD (SE)
Cost Coverage Waiting Time Public Provider Faith-Based Prov fhcorr*public perccorr*public HI Scheme 1	-0.00003 (5.18e-06)*** 0.36598 (0.01913)*** -0.00677 (0.0011)*** 0.07325 (0.03873)* 0.6919 (0.03505)*** -0.02934 (0.00718)*** 3.32962 (0.09704)***	0.39426 (0.02711)*** 0.03169 (0.001)*** 0.08236 (0.13421) 0.55507 (0.04972)*** 0.43805 (0.088)***	-0.00003 (5.18e-06)*** 0.36714 (0.01914)*** -0.0068 (0.0011)*** 0.11276 (0.06451)* 0.69372 (0.03517)*** -0.02054 (0.02681) 3.32085 (0.09699)***	0.39376 (0.02711)*** 0.0316 (0.00098)*** 0.07196 (0.13214) 0.565288 (0.0503)*** 0.01685 (0.05799)
No of households No of observations LR Chi2 (k-1) Prob > Chi2 (k-1) Log-likelihood	1458 39366 2373.56 0.0000 -9762.8307		1458 39366 2375.98 0.0000 -9761.4837	

Notes: SE stands for Standard Error. SD stands for Standard Deviation.

*** Significant at 99% confidence level

** Significant at 95%and * Significant at 90%.

The attributes of HIS have the expected effects. The attribute Cost has a negative sign, implying that the probability of participating in a HIS decreases as its cost rises. Coverage and faith-based provider are significantly and positively associated with the household’s likelihood to participate in the HIS. There is a strong preference in Sierra Leone for faith-based providers: they are perceived to be reliable because they have good doctors and are less plagued by corruption. There is also a significant positive effect on participating in a HIS if the provider is public. Nevertheless, experienced (and perceived) corruption reduces this effect significantly, which is captured by the negative sign of the measure of experienced (and perceived) corruption interacted with the HIS being provided by a public provider.

Using Table 6, we can compare the effect of experienced and perceived corruption on participation in a publicly provided HIS. The negative coefficients of both interaction terms fhcorr*pub and perccorr*pub indicate that households experiencing or perceiving higher corruption are less likely to participate in a publicly provided HIS than households that have not. The mean coefficient associated with fhcorr*pub is negative (-0.03) and significant, while the mean coefficient associated with perccorr*pub is also negative (-0.02) but not statistically significant. Otherwise, the two models show similar results: all other variables are statistically significant, and most coefficients exhibit substantial preference heterogeneity, as shown by their statistically significant standard deviations. In particular, the results indicate that, on average, the likelihood of households participating in a HIS is higher when the cost of the scheme and its waiting time are lower.

### Willingness to pay to participate in health insurance

The impact of corruption on households’ participation in a HIS can also be interpreted in terms of their WTP to participate if it is publicly provided. As explained in Section 2.5, we can use the coefficient of Cost to calculate the WTP for any other attribute by dividing the coefficient of interest by the negative of the cost coefficient (see [33] for a summary). Thus, in this case, the estimated WTP to participate in a publicly provided HIS is SLL 3,758 (i.e., 0.07325/0.00003) as per the Public Provider and Cost coefficients in the first model and SLL 2,441 (i.e., 0.11276/0.00003) as per the corresponding coefficients in the second one. Also, we can calculate the detriment in the WTP for a publicly provided HIS when the household has experienced (SLL -978) or perceived (SLL—685) corruption.

Table 7 shows that the WTP to participate in a publicly provided HIS decreases by 26% when the household has experienced corruption and decreases by 28% when the perception of corruption is high.

**Table 7:** WTP for a publicly provided HIS in the absence or presence of corruption.

	WTP for Public HIS when corruption is not present	Decrease in WTP for public HIS with experience of corruption	Total WTP for public HIS if corruption is present
Model 1—experienced corruption	3,758	-978	2,780
Model 2—perceived corruption	2,441	-684.67	1,756

Notes: The WTP figures are in the local currency—Sierra Leonean Leones (SLL).

## Discussion

Looking at our results, several points warrant discussion. Firstly, it was found that being more educated is associated with a higher probability of perceiving high levels of corruption and a lower likelihood of having experienced it. Intuitively, these heads of household are more aware of the corruption in the health sector and more able to protect themselves from it. Related to this, following the news is, as expected, associated with a higher likelihood of perceiving corruption, but surprisingly it does not protect individuals from experiencing it. Moreover, being in an urban setting is associated with a lower likelihood of perceiving corruption and a higher one of experiencing it. This is somewhat surprising as one would expect that living in cities facilitates communication, making citizens more aware of corruption and protecting them from experiencing it compared to more rural, more isolated areas. In this context, an explanation could be that there is a higher level of corruption in the healthcare sector in urban areas, which is not fully perceived by urban dwellers and affects them. Thirdly, we note that those in the Quarrying sector appear less likely to have experienced and perceived high levels of corruption than those in other sectors. A potential reason is that Quarrying is the smallest informal sector, with high poverty levels and living in unplanned settlements (i.e., also termed slums) [45]. This could mean less contact with the healthcare system and, thus, a lower likelihood of perceiving and experiencing corruption. Fourth, the negative relationship between the likelihood of both corruption measures and household distance to the health centre can be explained by the fact that increased distance results in reduced contact with healthcare providers. While increasing the likelihood of having experienced corruption, receiving remittances does not have a significant association with perceiving it. Finally, as expected, bad health is significantly associated with both measures: having suffered from malaria or typhoid fever might have exposed household members to seeking healthcare more intensively, and hence increased their likelihood of perceiving and experiencing corruption.

Our results further show that experienced corruption is a significant barrier to participation in HIS run by public providers. Also, from the results of the WTP analysis, we conclude that, although the WTP for a publicly provided HIS is positive according to our estimates, having experienced or perceived corruption in the health care system makes households’ WTP considerably less willing to pay for a HIS publicly provided, undermining their WTP by more than 25%. We interpret these results as suggesting corruption undermines trust in public institutions, including health care providers.

There are limitations to our study: although the information on the household we control for is quite extensive, there are unobservable factors that are not captured, for instance, the frequency and intensity of healthcare use. Healthcare usage can explain both the perception and the experience of corruption and, at the same time, the preferences of individuals for certain HISs.

## Conclusion

This paper studies the relationship between household characteristics and the likelihood of experiencing or perceiving corruption in the healthcare system in a low-income country such as Sierra Leone and how this affects the decision to participate in publicly provided HIS.

Although there is work on the determinants of joining health insurance schemes [47], there is very little done before on which households’ characteristics are correlated with the experience or perception of corruption in the health care sector of a low-income country. Our paper sheds light on this question in the specific context of workers in informal sector in Sierra Leone, who represent the majority of working adults in Sierra Leone. Second, we elicit households’ preferences over HIS’s attributes to investigate which ones incentivize and which ones discourage joining a HIS.

Our findings show that having experienced or perceived corruption in the health care system makes it less likely to participate in a publicly provided HIS, in line with previous literature [38]. Our results also corroborate previous findings showing that respondents are willing to pay significantly less for a HIS if they have experienced or perceived corruption [46].

From a methodological viewpoint, our results help explain why some previous studies often find lower-than-expected participation in HIS [26,47]. In these studies, revealed or observed participation in HIS might have suffered from the fact that perceptions of corruption were unobservable to the researcher. Our results show that perceived /experienced corruption explains a significant part of the variation in participation in a publicly provided HIS and the willingness to pay for it.

Finally, our paper contains a novel contribution to the existing literature on the WTP for health insurance–it estimates the extent to which corruption affects what households in the informal sector are willing to contribute to publicly provided health insurance schemes. Our findings are alarming: the WTP, when having experienced corruption, decreases by about a quarter.

This reveals the damaging spillover costs of corruption in the health care system in countries such as Sierra Leone. Not only corruption may make a health care system inefficient, but it also seems to undermine citizens’ trust in public institutions. These conclusions have strong implications for policymaking. They suggest that efforts to create HIS to provide health care services and essential medicines to those in need require to address the endemic corruption in healthcare provision.
